# In the flow of action: Anticipated action sequences in response-response binding

**DOI:** 10.3758/s13414-025-03154-y

**Published:** 2025-11-26

**Authors:** Maria Nemeth, Christian Frings, Birte Moeller

**Affiliations:** 1 Department of Psychology, Cognitive Psychology, Trier University, Trier, Germany; 2 Institute for Cognitive Affective Neuroscience (ICAN), Trier University, Trier, Germany

**Keywords:** Action control, Response-response binding, Planning of sequences, Anticipation

## Abstract

In theories of human action, it is assumed that individual actions are nested within higher-order action plans. This hierarchical structure oftentimes allows for the anticipatory planning of multiple future actions even before the current action is fully executed or situational cues demand this specific action. However, much of the existing research on basic action control processes has focused on isolated actions, that is, sequentially planned and executed actions, leaving it unclear whether these findings generalize to more naturalistic, preplanned action contexts. In particular, although the binding of individual responses into common representations and their retrieval from memory have been proposed as key mechanisms supporting action control of action sequences, it remains poorly understood how these processes operate when multiple responses can be planned in advance as part of an action sequence. In this study, we compared action contexts in which individual responses were planned and executed sequentially to contexts in which response sequences allowed for the preplanning of individual responses. Crucially, response-response binding effects of comparable strength were observed in both action contexts. Thus, binding and retrieval of responses seem not only to influence current performance during sequential action planning and execution but also to influence ongoing behavior within action sequences that could be preplanned.

## Introduction

Nearly all everyday actions consist of multiple components and can be considered sequential: from making a cup of tea, to preparing a meal, or even in music or sport, we frequently perform complex action sequences that require coordination of smaller, individual actions (e.g., Botvinick, 2008; Lashley, 1951; Miller et al., 1960). Crucially, every action is planned, at least to some degree, before its initiation (Rosenbaum, 1980) and action planning requires the selection of an action plan from a repertoire of several alternatives, which are is then organized into an intended and appropriate action sequence (cf. Jeannerod, 1997). However, when examining the literature on basic processes of action control, actions are typically studied in isolation. While this approach is useful for understanding the mechanisms supporting action control in a controlled manner, it leaves open the question of whether these processes can be directly applied to everyday situations involving anticipatory planning.

The literature on action control considers automated processes that closely link perception and action to ensure seamless coordination of behavior (Hommel, 1998). It is assumed that experienced features in the environment (objects, contexts) are bound to features of a (planned) action that occurs in close temporal contiguity, represented by the cognitive system through short-lived integrated feature-compounds in episodic memory, so-called event files (e.g., Frings et al., 2020, 2024; Hommel, 2004). When event file features are subsequently re-encountered in an action episode (e.g., a stimulus repeats), previously bound features are retrieved (including the action). This retrieval process allows for the automatic reactivation of the respective action without the need for (re-) computational processes of this action (Frings et al., 2020; Henson et al., 2014).

The processes of automatic feature binding and retrieval underlying human action control are typically investigated in *sequential* prime-probe paradigms (see Frings et al., 2020). It is assumed that responding to a stimulus in prime leads to binding of features of the stimulus and the response. Crucially, if a feature now repeats in probe (e.g., the stimulus repeats), the previously bound response is retrieved and affects current responding. That is, if the retrieved response matches the currently required probe response, performance is improved (e.g., faster reaction times (RTs) and lower error rates). On the other hand, if the stimulus retrieves a response that is incompatible with the response currently required, performance is impaired (e.g., slower RTs and higher error rates). This pattern of benefits and costs, depending on the compatibility of the retrieved and current action episode, is considered as evidence for binding between stimulus and response features (see the binding and retrieval in action control framework, BRAC; Beste et al., 2023; Frings et al., 2020).

Stimulus–response bindings are assumed to underlie most simple actions, supporting the automation of behavior (Frings et al., 2020; Hommel et al., 2001) and potentially playing an important role in habit formation (Giesen et al., 2020; Moeller & Pfister, 2022). However, recent evidence suggests that event codes can also represent *complex action sequences,* as demonstrated in bindings occurring between features of individual responses (response-response binding; Moeller & Frings, 2019a). In the response-response binding task, participants perform a sequence of four individually planned and executed actions. It is assumed that planning and executing two individual prime responses lead to their binding. If one of these responses is repeated in the following event (as probe response R1), it is theorized to trigger retrieval of the other bound response. If the retrieved response now matches the currently required response (probe response R2), performance is enhanced, and if they mismatch, performance is impaired (i.e., response-response binding effects).

Notably, in typical sequential binding tasks each stimulus that indicates a required response is presented individually. This ensures that responses are both planned and executed individually, avoiding potential overlap in the planning processes of these responses. By structuring tasks in this way, researchers can pinpoint incidental cognitive mechanisms of binding and subsequent retrieval of responses in action sequences. However, human action control appears way more complex than the sequential planning and execution of individual actions on their own (Kleijn et al., 2014): while we sometimes may plan actions only when a previous action was planned and executed, humans are capable of the planning and implementation of highly complex and coherent action sequences. That is, considering that individual actions are embedded in a continuous stream of behavior (e.g., Prinz, 2009; Stränger & Hommel, 1996), oftentimes the simoultaneous anticipation and coordination of multiple action goals is required (such as in sports, music, or even when making a sandwich).

In fact, it is widely accepted that action control is hierarchical, with individual actions being both composed of finer-grained actions and simultaneously contributing to the achievement of higher-level action goals (Moeller & Frings, 2019a, 2021b; see also Botvinick, 2008; Lashley, 1951; Miller et al., 1960; Norman, 1981; Prinz et al., 2013; Yamaguchi & Logan, 2014; Zacks & Swallow, 2007). Within these structures, the process of executing an individual action can also be influenced by the anticipation of a *subsequent* action plan. This is supported by studies showing that in goal-directed grasping movements, the hand begins to adjust before reaching the object, opening wider for larger objects (Jeannerod, 1981, 1994; for an overview of similar anticipatory effects in language, see Hommel, 2017).

Evidence for action anticipation also emerges in studies on more complex action sequences. It was demonstrated that bindings between response features can be (partially) established for actions that are planned but whose execution is temporarily postponed (e.g., Mocke et al., 2022; Stoet & Hommel, 1999; Wiediger & Fournier, 2008). In these experiments, participants planned an initial response for task A while withholding its execution until after planning and executing a subsequent response in task B. The typical finding is that performance in task B was impaired when its response features partially overlapped with those in task A, compared to when no features overlapped. These so-called partial repetition costs are further assumed to be contingent on the serial position of the overlapping feature code maintained in working memory, as they were only observed for the first of two action features (e.g., for the feature “left,” but not “up,” in the action plan “move left then up”; Fournier et al., 2014). Finally, evidence for the preplanning of entire action sequences comes from studies demonstrating that RTs decrease as fewer dimensions of the action sequence remain to be specified during execution (Rosenbaum, 1980; Rosenbaum & Saltzman, 1984).

However, it remains largely unexplored whether binding and retrieval of response features also support efficient action control in contexts in which entire response sequences comprising several individual responses can be preplanned as a whole. That is, in such a sequence, both the responses assumed to be bound and those that are potentially affected by these bindings could already be anticipated before initiation of the response sequence. Such a design allows us to examine how bindings are formed, retrieved, and affect subsequent performance within a sequence that could be fully prepared in advance. Crucially, such an action context contrasts with studies focusing on how the preplanning of an initial response (-sequence) modulates the execution of a second one that could not be anticipated at the time of planning the first response (e.g., Fournier et al., 2014). If binding and retrieval are fundamental processes in human action control (e.g., Frings et al., 2020; Hommel, 2004; Prinz, 1998), their influence should extend beyond paradigms in which each response is planned and executed strictly sequentially. An open question, however, is whether greater certainty about upcoming responses might alter the impact of these processes.

Consequently, if we assume that the same mechanisms of binding and retrieval are not only relevant for individual planning and execution as previously demonstrated (e.g., Frings et al., 2007; Moeller & Frings, 2019a), but also serve as highly adaptive and flexible processes in larger-scale action control, response-response bindings should influence current performance even when elements in response sequences are preplanned. Clarifying this question is crucial for understanding whether binding and retrieval serve as adaptive, flexible processes that coordinate not only isolated responses but also complex, preorganized action structures as they unfold in everyday behavior.

## The present study

The aim of the current study was to investigate binding and retrieval of responses in sequences that allow for the anticipation and therefore the preplanning of multiple actions within longer response sequences. To test this, we used a modified response-response binding task. In a sequential sequence information condition, we measured binding and retrieval of responses when responses were planned and executed individually. In a simultaneous sequence information condition, we measured binding and retrieval of responses when the entire sequence of responses could be anticipated before starting the initiation of the very first of the responses in the sequence.

We expected that if binding and retrieval of responses support hierarchical actions in longer sequences as previously assumed, response-response binding effects should influence current performance both when actions are planned and executed individually, and when actions are anticipated as part of an entire sequence. To anticipate the results, we found significant response-response binding effects emerging if responses were planned and executed sequentially as well as when the whole action sequence could be preplanned before response initiation.

### Method

#### Participants

Previous studies investigating response-response binding effects typically reported medium- to large-sized effects (e.g., Moeller & Frings, 2019a: *d* = 0.63 and *d* = 0.84). Thus, we were interested in at least a medium-sized response-response binding effect. The final sample consisted of 60 participants (41 female, 18 male, one diverse; 54 right-handers). The median age was 23 years (range: 19–34 years). A post hoc sensitivity analysis was conducted with the program G*Power (Faul et al., 2007) to determine the smallest detectable effect size with a sample size of 60, a significance level of α = 0.05 (one tailed), and a power of 1˗ß =.95. This analysis indicated that the experiment was powered to detect an effect size of at least *d* = 0.43.

Participants were recruited via Trier University’s participant platform (Sona Systems; sona-systems.com) and performed the experiment online on the experimental platform Pavlovia (Peirce & MacAskill, 2018). All participants consented via an online form before participating and received course credit as compensation. This study was carried out in accordance with the ethics guidelines declared by the ethics committee of Trier University. The ethics committee of Trier University declared all simple behavioral studies in accordance with their guidelines exempt from any further examinations by the committee.

#### Design

The design comprised three within-subjects factors, namely response R1 relation from prime to probe (response repetition vs. response change), response R2 relation from prime to probe (response repetition vs. response change) and sequence information (simultaneous vs. sequential).

#### Apparatus and stimuli

The experiment was programmed in PsychoPy (Peirce et al., 2019; Version 2023.2.3) and run online via Pavlovia (Peirce & MacAskill, 2018). Instructions were presented in white (according to the RGB color system: 255, 255, 255; font: Arial; font size: 20 pixels). Stimuli were shown in white or red (RGB: 255, 128, 128; font: Arial; font size: 25 pixels) on a gray background (RGB: 128, 128, 128). Stimuli were the digits 1, 2, 3, and 4 and the letters A, B, C, and D. Participants responded by pressing one of four keys (D, F, J, or K) on the computer keyboard. Additionally, the number “9” and the letter “P” indicated catch trials, which participants identified by pressing the “X” key on the keyboard, while non-catch trials had to be identified by pressing the space bar.

#### Procedure

Instructions were presented at the center of the screen. Participants were instructed to place their middle and index fingers of both hands on the keys D, F, J, and K. Their task was always to classify presented letters or numbers by pressing the corresponding key: using their left middle finger for the letter A and the number 1, their left index finger for B and 2, their right index finger for C and 3, and their left middle finger for D and 4. Participants were encouraged to respond as quickly as possible while also ensuring a high level of accuracy. The factor sequence information (simultaneous vs. sequential) was varied blockwise with one block in each of the two conditions. The order of blocks was counterbalanced across participants (note that block order did not affect any relevant effect^1^).

Each of the two experimental blocks (simultaneous vs. sequential) was preceded by a training session consisting of 16 trials: For participants who made more than 15% errors the training block was repeated (resulting in 32 training trials). In training, participants received performance-contingent feedback at the end of the trial both in the simultaneous condition and the sequential condition (for a correct response sequence: “correct sequence!”; for a wrong response: “wrong sequence!”).

**Fig. 1:**
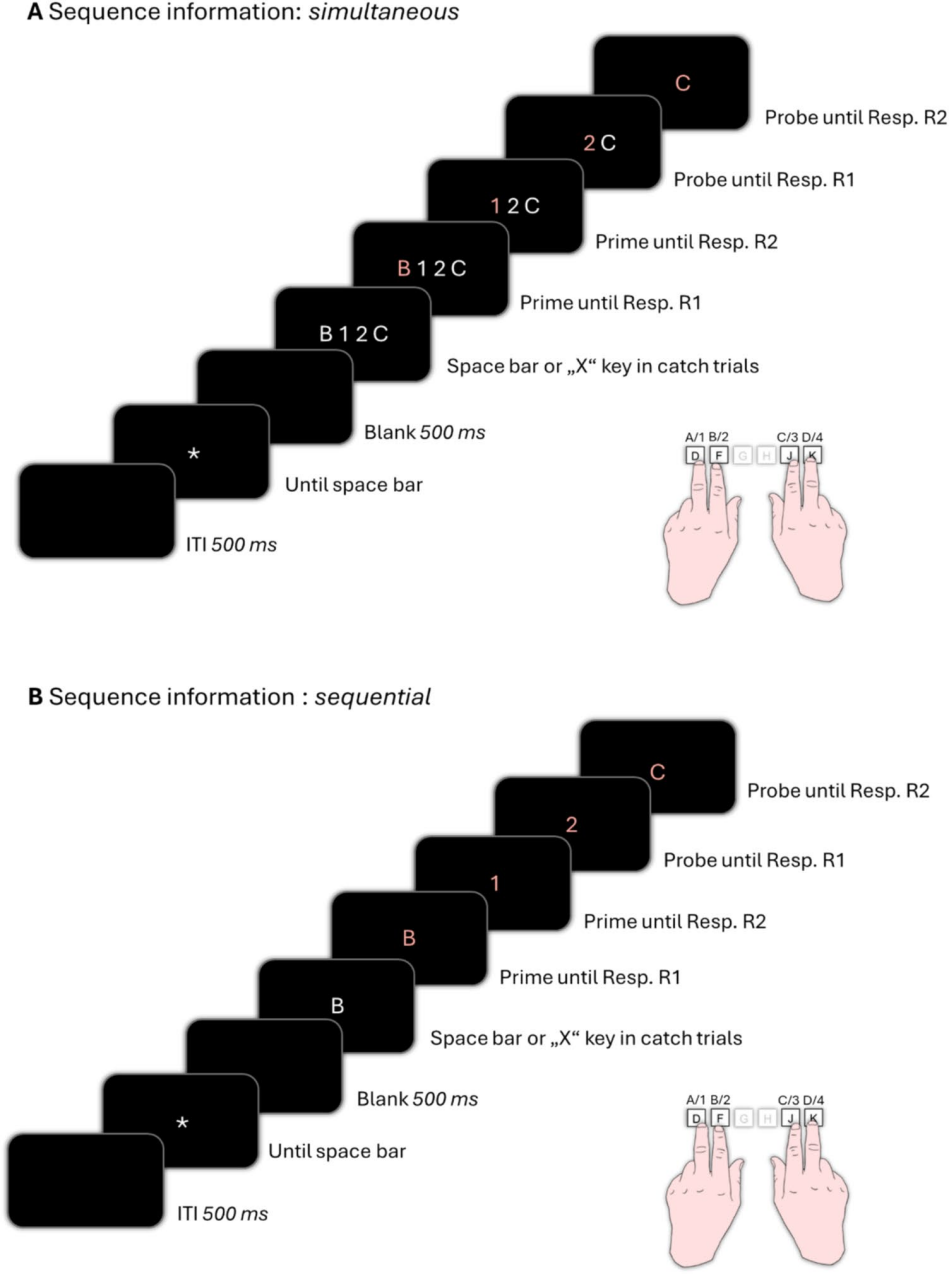
Sequence of events in one trial. Participants responded with their index and middle fingers of both of their hands to the identity of presented digits and letters. **A:** In the *simultaneous* sequence information condition, all in the trial to-be-responded-to stimuli were presented simultaneously at the beginning of the trial and then disappeared one by one after their corresponding response was executed. **B:** In the *sequential* sequence information condition, all stimuli were presented individually. A new trial was started by pressing the space bar. Stimuli are not drawn to scale. ITI: inter-trial interval

In the experimental block of the sequence information condition *simultaneous*, each trial started with an asterisk which indicated the space bar was to be pressed to start the trial (see Fig. 1A). A blank space appeared for 500 ms and was followed by the presentation of four stimuli in white horizontally aligned. In 20% of the trials, i.e., catch trials, one of the stimuli was a letter or a digit not assigned to one of the four critical response keys. Specifically, if the letter “P” or the number “9” appeared among the stimuli sequence, participants had to press the “X” key to indicate a catch trial. Once the “X” key was pressed, the trial immediately terminated, and feedback was given. For a correct identification of a catch trial the feedback “Correct, a P or a 9 was present, this is a catch trial!” was presented. If a catch trial was not correctly identified (i.e., the “X” key was not pressed) the trial terminated with the feedback “Wrong, a P/a 9 was present! In this case, always press the X on the keyboard!” In non-catch trials, where no catch stimuli appeared, participants had to press the space bar to proceed with the trial. After correctly pressing the space bar, the leftmost letter or digit turned red (while the other stimuli remained white), signaling that the corresponding response towards this stimulus is now to be carried out (prime response R1). After a response was detected, the red stimulus disappeared, so only the next three to-be-responded-to stimuli remained on the screen. After 300 ms, the first stimulus of the remaining sequence turned red, signaling that the response towards this stimulus was now to be carried out (prime response R2). Again, after a response was detected, the red stimulus disappeared, and the first stimulus of the remaining sequence turned red after 300 ms. After response detection of probe response R1 and the disappearance of the previous stimulus, the last remaining stimulus of the sequence turned red after 300 ms, indicating that the very last response (probe response R2) in this trial was now to be carried out. Participants received error feedback at the end of the trial, if any of the given responses in the trial was erroneous (“wrong sequence!”).

In the sequence information condition *sequential*, all stimuli were presented individually at the center of the screen. Each trial started with an asterisk indicating the space bar was to be pressed to start the trial (see Fig. 1B), followed by a 500-ms blank screen. The prime R1 stimulus was then presented in white. In 20% of the trials (catch trials), this stimulus was either “P” or “9,” requiring participants to press the “X” key. As in the simultaneous sequence information condition, performance-contingent feedback was provided for correct and missed catch trials. In non-catch trials, participants pressed the space bar to proceed. After correctly pressing the space bar in non-catch trials, the prime (R1) stimulus was presented in red, indicating that the prime response R1 was to be carried out. After a response was detected, a blank screen occurred for 300 ms. Then the second prime stimulus was individually presented in red, indicating prime response R2. After a response was detected, a blank screen occurred for 300 ms. Then the first probe stimulus was presented individually in red, indicating probe R1 until a response was detected, which was again followed by a blank space for 300 ms. Then the second probe stimulus appeared again individually in red, indicating probe R2 until response detection. Participants received error feedback at the end of the trial, if any of the given responses in the current trial was erroneous (“wrong sequence!”). Between the trials, a blank screen appeared for 500 ms (intertrial interval) before the asterisk indicated that the next trial could be started by pressing the space bar. Every 64 trials, participants were prompted to take a short break.

The factor relation of response R1 between prime and probe (repetition vs. change) was varied orthogonally. In R1 repetition trials, the same response required as prime response R1 was also required as probe response R1. In R1 change trials (R1c), a different response was required as prime response R1 and probe response R1. In R2 repetition trials (R2r), the identical response required as prime response R2 was also required as probe response R2. In R2 change trials (R2c), a different response was required as prime response R2 and probe response R2. For each response, whether a letter or a digit appeared as stimulus was randomly assigned. Importantly, stimuli did not repeat between prime and probe, thus a response repetition from prime to probe always involved a change from a letter to a digit or vice versa. Each experimental block included 256 trials (64 of each of the four conditions R1rR2r, R1rR2c, R1cR2r, R1cR2c).

### Results

Data processing and analysis were done with R (R Core Team, 2019; R Version 4.3.0). Performance in probe R2 was the dependent variable of interest. If the two responses R1 and R2 in the prime were integrated, repeating prime R1 as probe R1 should trigger retrieval of the second prime response R2, which would then influence probe R2. Crucially, in probe R2, participants responded significantly faster in the simultaneous condition (*M* = 176 ms, *SD* = 128 ms) compared to the sequential sequence information condition (*M* = 601 ms, *SD* = 90 ms), *F*(1, 58) = 1840.07, *p* <.001, ${{\eta }_{P}}^{2}$ =.97. To account for these overall RT differences between sequence information conditions, proportional RTs were computed by dividing RTs by the mean RT of each participant in each sequence information condition. This normalization procedure allowed for a more direct comparison of retrieval effects across sequence information conditions, independent of overall response speed differences.

Only trials with correct responses in both prime and probe were considered for the analysis of probe R2 RTs. Prime error rates were 1.8% for R1 and 2.1% for R2. Probe error rates were 2.9% for R1 and 2.5% for R2 (only including trials without errors in the previous responses). To analyze outliers in RTs, we followed the classification of Tukey (1977) and detected outliers using boxplots. Note that no participant had to be excluded due to being an outlier. On the participant's level, RTs that were more than 1.5 interquartile ranges above the third quartile of the participant’s RT distribution in each sequence information condition (Tukey, 1977) were excluded from the analyses. Due to these constraints, 2.2% of the trials were excluded from the RT analyses. See Table 1 for mean RTs and error rates in probe R2 performance.

**Table 1:** Proportional mean reaction times in ms and error rates in percent (in parentheses) for probe response R2, as a function of R1 relation from prime to probe, R2 relation from prime to probe and sequence information (simultaneous vs. sequential)

	Simultaneous		Sequential	
R1 Relation	R2 repetition	R2 change	R2 repetition	R2 change
R1 repetition R1 change	1.02 (2.4) 1.09 (4.0)	0.97 (1.8) 0.91 (0.86)	1.01 (2.5) 1.05 (4.4)	1.00 (2.4) 0.94 (1.9)

#### Probe R2 proportional reaction times (RTs)

**Fig. 2:**
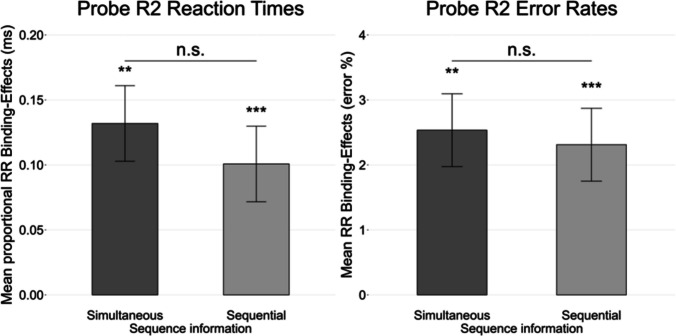
Response-response binding effects. Individual response-response binding effects in proportional reaction times and error rates as a function of sequence information for probe R2 performance. Response-response binding effects were calculated as the advantage of probe R1 repetition over probe R1 change in probe R2 repetition trials minus the advantage of probe R1 repetition over probe R1 change in probe R2 change trials: (R1cR2r-R1rR2r)-(R1cR2c-R1rR2c). The error bars depict the standard error of the mean. n.s. = *p* >.050, *p <.050, **p <.010, ***p <.001

A 2 (R1 relation: repetition vs. change) × 2 (R2 relation: repetition vs. change) × 2 (Sequence information: simultaneous vs. sequential) repeated-measure analysis of variance (ANOVA) on probe R2 RTs yielded a significant interaction between R1 relation and R2 relation, *F*(1, 58) = 25.23, *p* <.001, ${{\eta }_{P}}^{2}$ =.30, indicating response-response binding effects. The interaction between R1 relation and R2 relation and sequence information was not significant, *F*(1, 58) = 0.56, *p* =.455, ${{\eta }_{P}}^{2}$ <.01 (see Fig. 2). The individual response-response binding effects were significantly different from zero for the simultaneous condition (*M* = 0.13 ms, *SD* = 0.38 ms; *t*(59) = 3.12, *p* =.003, *d*z = 0.40) and the sequential condition *M* = 0.10 ms, *SD* = 0.09 ms; *t*(59) = 9.01, *p* <.001, *d*z = 1.16^2^). For the sake of completeness, the following other effects reached significance. The main effect of R2 relation was significant, *F*(1, 58) = 71.31, *p* <.001, ${{\eta }_{P}}^{2}$ =.55. Participants responded faster if the response changed from prime to probe (*M* = 0.96 ms, *SD* = 0.09 ms) than if it repeated (*M* = 1.04 ms, *SD* = 0.12 ms). The interaction of sequence information and R2 was significant, *F*(1, 58) = 6.00, *p* =.017, ${{\eta }_{P}}^{2}$ =.09. Follow-up analyses indicated faster RTs for response changes from prime R2 to probe R2 in the simultaneous sequence information condition compared to the sequential sequence information condition, *t*(59) = 2.52, *p* =.014, *dz* = 0.33, but slower RTs for response repetitions from prime R2 to probe R2 in the simultaneous sequence information condition compared to the sequential sequence information condition, *t*(59) = 2.34, *p* =.023, *dz* =.30. Finally, the interaction of block order and R1 relation reached significance, *F*(1, 58) = 5.96, *p* =.018, ${{\eta }_{P}}^{2}$ =.09. Follow-up analyses indicated faster RTs for response R2 repetitions in block order A (starting with the simultaneous block) compared to block order B (starting with the sequential block), *t*(238) = 1.98, *p* =.048, *dz* = 0.26, but no significant difference in RTs for response R2 changes between the block orders A and B, *t*(238) = 1.55, *p* =.124, *dz* = 0.20. None of the other effects reached significance, *F*s < 4, *p*s >.06, ${{\eta }_{P}}^{2}$ <.06.

#### Probe R2 error rates

In the same analysis on error rates, the interaction between R1 relation and R2 relation was significant, *F*(1, 58) = 21.48, *p* <.001, ${{\eta }_{P}}^{2}$ =.27, indicating response-response binding effects. The interaction between R1 relation and R2 relation and sequence information was not significant, *F*(1, 58) < 1, *p* =.780, ${{\eta }_{P}}^{2}$ <.01 (see Fig. 2). The individual response-response binding effects were significantly different from zero for the simultaneous condition (*M* = 2.5%, SD = 4.8%, *t*(59) = 3.38, *p* =.001, *d*z = 0.44), and sequential condition (*M* = 2.3%, SD = 5.3%, *t*(59) = 4.10, *p* <.001, *d*z = 0.53).

For the sake of completeness, the main effect of R1 relation was significant, *F*(1, 58) = 4.08, *p* =.048, ${{\eta }_{P}}^{2}$ =.07. Participants made fewer errors if the response repeated from prime to probe (*M* = 2.3%, *SD* = 2.6%) than if it changed (*M* = 2.8%, *SD* = 3.6%). Finally, the main effect of R2 relation was significant as well, *F*(1, 58) = 30.92, *p* <.001, ${{\eta }_{P}}^{2}$ =.35. Participants made fewer errors if the response changed from prime to probe (*M* = 1.7%, *SD* = 2.3%) than if it repeated (*M* = 3.3%, *SD* = 3.7%).

#### Additional analyses

In addition to the analysis of probe 2 performance, the simultaneous planning condition may have allowed preplanning of the response sequence as a whole, thereby potentially allowing binding and retrieval to influence earlier responses than probe 2 response. To account for this, we also analyzed performance in probe R1, prime R2, and prime R1 responses. For all those dependent variables, proportional RTs were used in order to control for general RT differences between sequence information conditions (see Appendix A for the corresponding analyses).

##### Probe R1 performance

A 2 (R1 relation: repetition vs. change) × 2 (R2 relation: repetition vs. change) × 2 (Sequence information: simultaneous vs. sequential) repeated-measure analysis of variance (ANOVA) on probe R1 proportional RTs yielded a significant interaction between R1 relation and R2 relation, *F*(1, 58) = 6.10, *p* =.017, ${{\eta }_{P}}^{2}$ =.10, indicating response-response binding effects. The interaction between R1 relation and R2 relation and sequence information was significant, *F*(1, 58) = 6.45, *p* =.014, ${{\eta }_{P}}^{2}$ =.10, indicating stronger response-response binding effects in the simultaneous condition compared to the sequential condition. The individual response-response binding effects were significantly different from zero for the simultaneous condition (*M* = 0.06 ms, SD = 0.18 ms, *t*(59) = 2.64, *p* =.010, *dz* = 0.34) but not for the sequential condition (*M* = 0.01 ms, SD = 0.06 ms, *t*(59) = −0.70, *p* =.489, *dz* = −0.09). For the sake of completeness, the main effect of R1 relation was significant as well, *F*(1, 58) = 40.02, *p* <.001, ${{\eta }_{P}}^{2}$ =.40. Participants responded faster if the response changed from prime to probe (*M* = 0.97 ms, *SD* = 0.07 ms) than if it repeated (*M* = 1.03 ms, *SD* = 0.07 ms). None of the other effects reached significance, *Fs* < 2, *ps* >.3, ${{\eta }_{P}}^{2}$ <.02.

In the same analysis on error rates, the main effect of R1 relation was significant, *F*(1, 58) = 13.00, *p* <.001, ${{\eta }_{P}}^{2}$ =.18. Participants made fewer errors if the response changed from prime to probe (*M* = 2.3%, *SD* = 2.5%) than if it repeated (*M* = 3.0%, *SD* = 3.2%). The interaction between R1 relation, sequence information and block order was significant, *F*(1, 58) = 4.08, *p* =.048, ${{\eta }_{P}}^{2}$ =.07. None of the other effects reached significance, *F*s < 4, *p*s >.06, ${{\eta }_{P}}^{2}$ <.06. See Table 2 for mean RTs and error rates in probe R1 performance.

**Table 2:** Proportional mean reaction times in ms and error rates in percent (in parentheses) for probe response R1, as a function of R1 relation from prime to probe, R2 relation from prime to probe and sequence sequence information (simultaneous vs. sequential)

	Simultaneous		Sequential	
R1 Relation	R2 repetition	R2 change	R2 repetition	R2 change
R1 repetition R1 change	1.02 (2.8) 0.99 (2.3)	1.04 (2.6) 0.95 (2.3)	1.02 (3.4) 0.97 (2.4)	1.03 (3.5) 0.98 (2.0)

##### Prime R2 performance

A 2 (R1 relation: repetition vs. change) × 2 (R2 relation: repetition vs. change) × 2 (Sequence information: simultaneous vs. sequential) repeated-measure analysis of variance (ANOVA) on prime R2 proportional RTs yielded no significant effects, *Fs* < 2, *ps* >.2, ${{\eta }_{P}}^{2}$ <.03. In the same analysis on error rates, the main effect of sequence information was significant, *F*(1, 58) = 22.09, *p* <.001, ${{\eta }_{P}}^{2}$ =.28. Participants made fewer errors in the simultaneous condition (*M* = 1.3%, *SD* = 1.7%) compared to the sequential condition (*M* = 2.5%, *SD* = 2.9%). None of the other effects reached significance, *Fs* < 4, *ps* >.07, ${{\eta }_{P}}^{2}$ <.06 (see Appendix B, Table 3 for mean RTs and error rates in prime R1 performance).

##### Prime R1 performance

A 2 (R1 relation: repetition vs. change) × 2 (R2 relation: repetition vs. change) × 2 (Sequence information: simultaneous vs. sequential) repeated-measure analysis of variance (ANOVA) on prime R1 proportional RTs yielded no significant effects, *Fs* < 3, *ps* >.1, ${{\eta }_{P}}^{2}$ <.04. In the same analysis on error rates, the main effect of sequence information was significant, *F*(1, 58) = 7.80,* p* =.007, ${{\eta }_{P}}^{2}$ =.12. Participants made fewer errors in the sequential condition (*M* = 1.0%, *SD* = 1.6%) compared to the simultaneous condition (*M* = 1.5%, *SD* = 2.1%). None of the other effects reached significance, Fs < 4, ps >.05, ${{\eta }_{P}}^{2}$ <.07 (see Appendix C, Table 4 for mean RTs and error rates in prime R1 performance).

## General discussion

The present study investigated whether binding and retrieval mechanisms facilitate ongoing performance when (elements in) action sequences can be preplanned, also allowing for the simultaneous planning of these elements. While previous research has emphasized that complex action sequences are at least partially planned before their initiation, the role of binding and retrieval in such anticipatory planning contexts has remained largely unexplored. Addressing this gap, the present study examined binding and retrieval of responses within action sequences, comparing conditions under which participants either planned and executed individual responses sequentially or were able to preplan multiple elements of an upcoming action sequence.

To address this question, we employed an adapted response-response binding paradigm that differed from the traditional task in some respects. In a sequential planning condition, the procedure was identical to the classic paradigm except for two differences. That is, prime and probe responses were not separated by a response-stimulus interval (typically 500 ms in the standard task; Moeller & Frings, 2019a, 2019b), and feedback was provided only at the end of the trial rather than immediately after erroneous responses. Importantly, previous work demonstrated that response-response binding effects are not dependent on a time interval separating prime and probe responses (Moeller et al., 2025; Nemeth et al., 2025) and that robust, comparable binding and retrieval effects can be observed regardless of whether feedback for erroneous responses is provided (Foerster et al., 2023). In a simultaneous planning condition, by contrast, response-associated stimuli for the entire trial were already presented before the execution of the first response. Thus, while the sequential condition closely mirrored traditional response-response binding procedures apart from the absence of a response-stimuli interval and delayed feedback, the simultaneous planning condition allowed participants to preplan the responses within the trial.

Importantly, two observations suggested that participants did indeed engage in advance planning in the simultaneous condition. First, we observed response-response binding effects already in probe R1 performance when preplanning was possible (i.e., the three-way interaction between R1 relation, R2 relation, and sequence information). This pattern of results clearly indicated that, at the moment of executing probe R1, both probe responses were already represented, creating interference with responses bound during the prime. One plausible explanation of this observed effect is that both probe responses were anticipated prior to probe R1 execution and that this anticipation of the probe R2 retrieved (when matching with the prime R2) the bound prime R1, which could either facilitate or hinder probe R1 execution. Alternatively, one might speculate if the anticipation of the probe R1 triggered prime R2 retrieval, and that the anticipation of probe R2 as a matching or mismatching response compared to the retrieved prime R2 already influenced probe R1 responding (instead of probe R2 responding, as usually observed). Although our findings do not allow firm conclusions to be drawn about the temporal order of activation and retrieval, they support the idea that retrieval can be triggered by the mere planning of a response (Nemeth et al., 2024) and extends it by suggesting that anticipation of a subsequent response can modulate the execution of a current response. In addition, we observed significantly shorter RTs for responses within the trial in the simultaneous planning condition compared to the sequential planning condition. Such reductions in RTs have previously been attributed to the predictability of action elements within a sequence, allowing for optimized control processes (see, e.g., Willingham et al., 1989). Thus, we assume that individual actions were initiated more rapidly due to earlier preplanning.

Across both planning conditions, we observed robust response-response binding effects in probe R2 responses (as traditionally investigated). Notably, the magnitude of these effects did not differ between the two planning conditions, suggesting that effects of feature binding and retrieval are similarly important for current performance, whether actions are planned step-by-step or in advance. Thus, while earlier studies demonstrated that sequentially planned and executed actions can become bound, thereby representing higher-order representations of individual actions in action sequences (e.g., Moeller & Frings, 2019a), the present findings demonstrate that individual planning is not a prerequisite for the formation of these bindings. Thereby, our results increase the scope of event-coding approaches (Frings et al., 2020; Hommel et al., 2001) to situations in which response-sequences can be completely preplanned before the sequence starts.

In addition to the effects central to our hypothesis, several further results warrant brief discussion. We observed generally faster probe R2 RTs for R2 response changes from prime to probe compared to R2 response repetitions. That is, we can assume that when responding to a stimulus, stimulus features are bound with features of the response (see Moeller & Frings, 2021a). When, in a subsequent trial, a different stimulus is presented but requires the same response (as it is typical in the response-response binding task including response repetitions without stimulus repetitions), this constitutes a partial feature repetition, since stimulus features change but response features repeat. Such partial feature repetitions are assumed to disrupt performance, because the previously formed event file interferes with the current one (the same logic applies to the main effects of R1 relation and R2 relation we found in error rates). This effect was further modulated by sequence information, suggesting that the difference between R2 response repetitions and changes was even larger when both stimuli were presented simultaneously (i.e., when the response sequence was predictable) compared to when they were presented sequentially.

That binding and retrieval indeed do operate in action sequences allowing for the preplanning of actions is particularly relevant given the anticipatory nature of human action control. Rather than planning and executing actions step-by-step, actions are assumed to be embedded in higher-order action plans that allow for the anticipation and partial preparation of future steps (e.g., Botvinick & Plaut, 2004; Rosenbaum et al., 1983). This anticipatory nature in action control has long been emphasized: on a micro-level, individual actions are assumed to be initiated by anticipating their action effects (e.g., Kunde, 2001), while on a more macro-level, anticipating future actions allows for the planning/programming of action elements that facilitate the initiation and execution of complex action sequences (e.g., Rosenbaum, 1980; Rosenbaum & Saltzman, 1984; Rosenbaum et al., 1984). Our results align with these previous findings on the preplanning of action sequences by showing that once the response sequence was initiated, individual responses were executed faster when participants could anticipate the action sequence in advance, compared to when the task only allowed planning and executing actions individually. This fits the assumption that action planning is not a temporally discrete process restricted to the very moment before its implementation. Rather, action plans are thought to unfold over time in the course of both simple actions and complex action sequences, and were defined as everything that occurs between setting an intention and the final neural signal that triggers muscle contraction (Rosenbaum, 2013). Furthermore, the finding that action sequences can be initiated before all sequence elements are fully planned (in terms of Hommel et al., 2001) or programmed (in terms of Rosenbaum et al., 1984) point to the possibility that action planning and initiation are independent processes (for an overview, see Hommel, 2017). Against this background, the current finding provides compelling evidence that binding and retrieval are robust mechanisms even in situations involving overlapping planning and execution processes, supporting the flexible and efficient action control of complex, hierarchically structured action plans that better reflect the complexity of real-world action control.

It must be noted that we did not observe response-response binding effects in the prime R1 and prime R2 responses. Crucially, binding of action sequences consisting of two movements were already demonstrated during planning (e.g., Fournier et al., 2014). Consequently, if we assume that responses within the entire action sequence can be preplanned (i.e., be integrated) and may even be retrieved prior to the action sequence’s initiation, one could expect binding effects to already manifest in early responses of the sequence (i.e., prime R1 or R2). However, previous studies demonstrated that response-response binding effects are reduced if either the to-be-bound or the retrieving response within a sequence is omitted (Nemeth et al., 2024). Together, these findings suggest that while planning alone may be sufficient to trigger the activation of the cognitive representation of an individual response, merely preparing an entire sequence of multiple responses does not seem to create sufficient activation for binding and subsequent retrieval at the planning level. In other words, whether partial or complete repetitions/changes occur during the planning of a response sequence appears not to influence how easily the respective sequence is initiated. Rather the motor execution of responses seems to play a crucial role in determining the extent to which binding and retrieval affect performance. This interpretation is in line with the assumption that only some components of an action plan within a sequence are fully specified in advance, whereas other aspects are refined as execution unfolds of action plans later on as they become clear during execution (e.g., Rosenbaum et al., 1987). This view is consistent with the assumptions of the Theory of Event Coding (Hommel, 2009; Hommel et al., 2001), which proposes that high-level processes specify goal-relevant action characteristics in advance, whereas the final specification of open parameters is completed during execution through the weighting of task-relevant stimulus dimensions. Such “completion” processes may therefore be essential for binding and retrieval to exert their full influence within action sequences.

Rather, our findings suggest that simultaneous anticipation enables retrieval not only to produce the usual effects from previously executed responses but also to allow the immediately upcoming response to influence the execution of the current response. This suggests that retrieval processes can flexibly integrate both past and next elements within a preplanned sequence, whereas responses that lie further ahead in the sequence do not appear to exert such an influence.

Furthermore, the assumption that sequential actions are represented hierarchically rather than sequentially raises the question of whether an action sequence may be planned as a single, larger-scale action. Future research should further investigate how binding mechanisms support the formation of hierarchical action structures, for example, if event files representing entire action sequences on a larger scale can influence the initiation and execution of a following action sequence.

Taken together, our findings support the view that bindings can extend beyond isolated stimulus–response events to represent individual actions in higher-order representations (Moeller & Frings, 2019a). Interestingly, our results suggest that individual planning is not a prerequisite for the formation of these higher-level representations. Instead, the cognitive system appears capable of flexibly integrating individual actions into common event representations, even when these actions are preplanned as part of a coherent action sequence. This is in line with theoretical assumptions that binding and retrieval not only affect behavior if they function sequentially (i.e., in sequential tasks), but in virtually all situations allowing for intertwined action planning and execution processes in hierarchical action control. This considerably broadens the range of contexts in which binding and retrieval processes contribute to successful action control, including more naturalistic situations where longer action sequences must be initiated and coordinated flexibly based on anticipatory knowledge of individual subordinate actions. For example, when operating modern technological devices, users often have to perform sequences of actions that are guided by higher-level goals and mediated via user interfaces, requiring integration of motor actions, visual feedback, and anticipatory planning in highly dynamic contexts (e.g., for a computational model of perception and action in human computer interaction, see Haazebroek & Hommel, 2009).

## Conclusion

The present study demonstrates that binding and retrieval effects are not confined to stepwise action execution but are robust and operate similarly when elements of an action sequence are preplanned. Under conditions that allow for such preplanning, our findings suggest that the anticipation of a later response, depending on whether it matches or conflicts with the contents of the retrieved event file, can modulate the execution of the current response that triggered retrieval. By showing that binding and retrieval operate within preplanned action sequences, the present research extends current theories of action control and provides new insights into how action anticipation supports efficient coordination of future actions.

## Data Availability

The data for the experiment are available at OSF (https://osf.io/32rb6/?view_only=d1092c21a6c045b8add6066dee80790b), we here provide trial-level and subject-level data for the conducted experiment. None of the experiments were preregistered.

## Appendix A

As for probe R1 performance, proportional reaction times (RTs) were used within sequence information conditions because participants responded significantly faster in the simultaneous condition compared to the sequential condition (simultaneous condition: *M* = 261 ms, *SD* = 168 ms; sequential condition *M* = 607 ms, *SD* = 88 ms; *F*(1, 58) = 700.00, *p* <.001, ${{\eta }_{P}}^{2}$ =.92). For prime R2 performance, proportional RTs were used as well (simultaneous condition: *M* = 195 ms, *SD* = 124 ms; sequential condition *M* = 583 ms, *SD* = 87 ms; *F*(1, 58) = 1398.14, *p* <.001, ${{\eta }_{P}}^{2}$ =.96). For prime R1 performance, participants responded significantly slower in the simultaneous condition (*M* = 485 ms, *SD* = 155 ms) than in the sequential condition (*M* = 313 ms, *SD* = 109 ms), *F*(1, 58) = 132.08, *p* <.001, ${{\eta }_{P}}^{2}$ =.69). Therefore, again proportional RTs were used.

## Appendix B

ANOVAs on Prime 2 proportional RTs and error rates.

**Table 3:** Proportional mean RTs in ms and error rates in percent (in parentheses) for prime response R1, as a function of R1 relation from prime to probe, R2 relation from prime to probe and sequence information (simultaneous vs. sequential)

	Simultaneous		Sequential	
R1 Relation	R2 repetition	R2 change	R2 repetition	R2 change
R1 repetition R1 change	0.99 (1.2) 1.00 (1.3)	1.01 (1.3) 1.00 (1.1)	1.00 (2.8) 1.00 (2.6)	1.00 (2.6) 1.00 (2.0)

## Appendix 3

ANOVAs on Prime 1 proportional RTs and error rates.

**Table 4:** Proportional mean RTs in ms and error rates in percent (in parentheses) for prime response R1, as a function of R1 relation from prime to probe, R2 relation from prime to probe and sequence information (simultaneous vs. sequential)

	Simultaneous		Sequential		
R1 Relation	R2 repetition	R2 change	R2 repetition	R2 change	
R1 repetition R1 change	1.00 (1.9) 1.00 (1.3)	0.99 (1.3) 1.00 (1.7)	1.00 (0.9) 1.00 (1.0)		1.01 (1.2) 1.00 (1.2)

## Footnotes

1 *Block Order A*: (1) Simultaneous, (2) Sequential; *Block Order B*: (1) Sequential, (2) Simultaneous.

2 Note that although the mean binding effect was descriptively larger in the simultaneous condition compared to the sequential condition, the larger standard deviation in the simultaneous condition resulted in a smaller standardized effect size.
